# Medical students as global citizens: a qualitative study of medical students’ views on global health teaching within the undergraduate medical curriculum

**DOI:** 10.1186/s12909-019-1631-x

**Published:** 2019-05-30

**Authors:** Nicole Blum, Anita Berlin, Anna Isaacs, William J. Burch, Chris Willott

**Affiliations:** 1 Development Education Research Centre, UCL Institute of Education, 36 Gordon Square, London, WC1H 0PD UK; 2 Barts & the London School of Medicine, Queen Mary University of London, London, UK; 3 Centre for Food Policy, City, University of London, London, UK; 4 Richmond Pharmacology, London, UK; 5 Centre for Global Health and Health Partnerships, King’s College London, London, UK

**Keywords:** Global health, Pedagogy, Student perspectives

## Abstract

**Background:**

There is increasing interest in global health teaching among medical schools and their students. Schools in the UK and internationally are considering the best structure, methods and content of global health courses. Academic work in this area, however, has tended to either be normative (specifying what global health teaching *ought* to look like) or descriptive (of a particular intervention, new module, elective, etc.).

**Methods:**

While a number of studies have explored student perspectives on global health teaching, these have often relied on tools such as questionnaires that generate little in-depth evidence. This study instead used qualitative methods to explore medical student perspectives on global health in the context of a new global health module established in the core medical curriculum at a UK medical school.

**Results:**

Fifth year medical students participated in a structured focus group session and semi-structured interviews designed to explore their knowledge and learning about global health issues, as well as their wider perspectives on these issues and their relevance to professional development. While perspectives on global health ranged from global health ‘advocate’ to ‘sceptic’, all of the students acknowledged the challenges of prioritising global health within a busy curriculum.

**Conclusions:**

Students are highly alert to the diverse epistemological issues that underpin global health. For some students, such interdisciplinarity is fundamental to understanding contemporary health and healthcare. For others, global health is merely a topic of geographic relevance. Furthermore, some students appeared to accept global health as a specialist area only relevant to professionals working overseas, while others considered it to be an essential part of working in the globalised world and therefore relevant to all medical professionals. Students also clearly noted that including ‘soft’ subjects and more discursive approaches to teaching and learning often sits awkwardly in a programme where ‘harder’ forms of knowledge and didactic methods tend to dominate. This suggests that more work needs to be done to explain the relevance of global health to medical students at the very beginning of their studies.

**Electronic supplementary material:**

The online version of this article (10.1186/s12909-019-1631-x) contains supplementary material, which is available to authorized users.

## Background

Global health is an area of increasing interest for educators and students of medicine [1, 2], with growing numbers of medical schools offering global health teaching [3] and in the context of urgent arguments for its relevance to professional practice [4]. Most medical schools offer global health in the form of optional modules, overseas electives, or intercalated degrees, however, rather than as a part of the core curriculum to be studied by all medical students as recommended by Rowson et al. [5]. Recent interest results from a recognition of the globalised nature of medical practice, related to high profile cross border health issues (e.g., swine flu), increasing rates of migration (both of patients and healthcare professionals), and the resulting ethnic diversity in the so-called global north [3, 5, 6].

A number of paradigms influence the choices educators need to make in designing an appropriate curriculum and debate continues both as to how global health should be defined as well as what a successful teaching programme looks like [3, 5]. The established paradigm of international health focuses on the health needs of individuals in ‘other’ countries, for instance, and stands in contrast to approaches to global health which consider health, healthcare and health inequalities as transnational phenomena in a globalised context [5]. The shift in focus from *international* to *global* health is also reflected in debates in higher education around internationalisation of programmes and global citizenship of students and faculty [7, 8]. Given the increasingly interconnected nature of local and global health issues, it is important for medical schools to ask what global health competencies students require [6, 9, 10].

Global health is included in licensing outcomes in the UK [11], although these lack detail regarding content. Johnson et al. [10] attempted to address this for the UK context, and similar discussions about standardisation have taken place in the USA and Canada [12, 13]. A recent literature review [14] noted that despite an apparent consensus, however, implementation has been patchy.

This delay might be explained by the conflicting discourses embedded in rationales for making global health a core medical competency. In a review of over a thousand publications, Martimianakis and Hafferty [6] identified three separate discourses: the universal physician (who can practise anywhere); the culturally versed physician (whose medical knowledge is culturally informed locally and internationally); and the global physician advocate (socially minded and informed regarding the economic, political and cultural determinants of health and equipped to use their status to advocate for global and local policy and populations). These discourses echo Rowson at al’s three types of doctor: the ‘globalised doctor’, the ‘humanitarian doctor’ and the ‘policy doctor’ [5]. Both papers conclude that these essentially separate discourses or professional identities are associated with different pedagogic focus and methods.

Murdoch Eaton et al. [3] outline three pedagogic approaches which can underpin teaching in medical schools: ‘additive’, where global health teaching is an addition to the main curriculum (optional); ‘integrated’, where teaching is embedded into the mainstream curriculum, and ‘transformative’, in which teaching is ‘embedded throughout the programme, with a dynamic and interactive effect on both’ [3]. Overall, however, in-depth research on global health teaching is limited. Studies tend to either be normative, by exploring what global health teaching *ought* to look like [3, 12, 15], or descriptive, by detailing a particular intervention, new module, or elective [1, 16]. Much of this literature is specifically related to international medical electives [17–21]. One recent review noted that medical schools showed no consensus on either the competencies required or optimal teaching methods, with many papers merely making recommendations for the future rather than detailing current approaches [16].

A number of papers consider the student perspective [2, 9, 22], but tend to be aspirational, generally promoting a curriculum that develops competencies aligned to the advocacy physician or humanitarian doctor with little description, evaluation or critical analysis of actual interventions and their impact on the wider student body. A number of post-course evaluations also focus on overseas electives using quantitative tools [9, 13, 22, 23].

Gaining a more in-depth understanding of students’ perceptions was therefore a key aim of this research, both because of the relative lack of research in this area, as well as our own experiences. Both evidence (unpublished) from student evaluations that the authors have received, as well as a number of years of involvement in global health teaching and learning suggested that while a minority of students strongly support the inclusion of international and global aspects of health in their course (we estimate approximately a quarter), the majority of students are either indifferent (around half) or consider the topics unimportant to them as professionals, and with no legitimate place in the medical programme (approximately a quarter). We therefore used this broad framework within the research design, and refer to these subgroups in the following paper (rather simplistically) as advocates, uncertain or sceptics.

### Context – Introducing a new Global Health module

At the time of this research, global health was included in UCL Medical School’s curriculum in the following ways: optional student selected components (SSCs) (years 2 or 6); an integrated BSc programme (year 3); and the Global Health module (year 5). The mandatory academic components of the Student Elective (taken abroad by over 80% of final years) are also part of the global health curriculum. These include a pre-elective study of the site/ country to be visited (including the UK, if appropriate) and a post-elective written report.

The year 5 Global Health module is the educational intervention that is focus of this article. The module is what Murdoch Eaton et al [3] call ‘integrated’, in that it is a compulsory, but standalone module (see Table 1) which two of the authors of this paper (AB and CW) were involved in designing. The time and faculty available for the module were limited. The content, learning goals and teaching methods were therefore chosen to best use limited time to equip students with basic knowledge and clinically-oriented analytical skills. Recognising global health is a field that draws on numerous disciplinary traditions, we chose a mix of pedagogic strategies to expose students to key factual sources as well as to provide opportunities to integrate knowledge and to debate complex issues through case studies. We also hoped that this mix of content and methods would engage a wider range of students.

**Table 1:** Overview of the Global Health Module (Year 5)

Element	Title	Teaching Methods
Session 1	Planning Your Elective	Lecture
Session 2	Gender and Global Health	Lecture
Session 3	Health Systems and Burden of Disease	Lecture
Session 4	Global Health Dilemmas and the Student elective	Facilitated group tutorials based on anonymised case studies
Session 5	The Global is Local: Healthcare of migrants, refugees and the undocumented in London	Lecture followed by facilitated group tutorials based on anonymised case studies
Session 6	‘Ebola: The UK response to a global public health emergency’	Lecture
Assessment	Pre Elective Country Study	Written analysis of personal learning goals, anticipated challenges, healthcare and burden of disease (25,00 words) added to Student Portfolio
Assessment	End of Year Exam	Single Best Answer questions

## Methods

This small scale qualitative study was carried out in the academic year 2014–2015. Ethical approval for the project was granted by the UCL Research Ethics Committee prior to any data collection. The module described in Table 1 was used as a trigger for the research, but what is presented here is not a formal evaluation. Rather, it was an exploratory qualitative study which three of the authors (NB, AB and CW) designed in order to explore Year 5 medical students’ knowledge and learning about global health issues, as well as their wider perspectives on its relevance to their professional development. The second of these aims is the focus of the following discussion.

The first phase of the study took place at the beginning of the module and comprised a structured one-hour focus group session held in September 2014. This was followed by individual semi-structured interviews of approximately 30 min each in late October 2014. In the second phase, which took place after the module had finished, students participated in a further follow up individual interview between mid-December 2014 and mid-January 2015. The aim of the second series of interviews was to ask students to reflect on the module as a whole (e.g. topics covered, teaching approaches), but also provided an opportunity for further discussion of the topics raised in the focus group and earlier interviews (see Additional files 1 and 2 for focus group session plan and interview guides). The corresponding author (NB), who did not previously know any of the participants, conducted both the focus group and interviews. The focus group took place on the UCL campus and interviews were conducted by telephone.

The year 5 cohort at the time had 360 students in total, with between 80 and 90% attendance recorded for global health lecture sessions and between 70 and 80% for global health workshops over the year. It is important to note here that while the authors who designed the study (NB, AB and CW) had initially hoped for a larger group of study participants, this proved difficult to achieve given the demanding schedules of medical students. As a result, the final study included a small and self-selected sample of volunteer respondents.

Initial recruitment for the study was carried out at the end of a lecture which was part of the compulsory Year 5 Global Health module (Session 3; see Table 1). One of the authors (AB) provided a brief overview of the planned research and asked students to volunteer to take part in a short focus group discussion. While a formal count was not made of those in attendance on the day, the majority of the cohort was present. Of those in attendance, seven students agreed to participate in the focus group. At the beginning of the session, the students were provided with a more detailed overview of the study and its aims, and assured of the confidentiality and anonymity of any data collected during the session. They were also asked to describe any previous experiences of global health and to identify themselves along a spectrum of interest, ranging from ‘advocate’ to ‘uncertain’ to ‘sceptic’. Of the group, three students self-identified as global health ‘advocates’, three self-identified as ‘uncertain’, and one self-identified as a ‘sceptic’ (see Table 2 below). At the end of the focus group session, the participants were asked if they would also be willing to take part in the follow up one-on-one interviews. Five of the students agreed to participate in this initial series of interviews, and three of those also took part in the second round of interviews after the module had finished.

**Table 2:** Participant details

ID Number	Gender	Self-Identified Category	Previous experience of global health	Focus Group	Interview 1	Interview 2
S1	F	*uncertain*	none	X	X	X
S2	M	*uncertain*	volunteer special police constable; BSc in Primary Care and Public Health	X	X	
S3	M	*sceptic*	none	X	X	
S4	M	*uncertain*	Year 2 SSC in Global Health	X	X	X
S5	M	*advocate*	BSc in Global Health	X		
S6	F	*advocate*	BSc in Global Health	X		
S7	F	*advocate*	active participant in the UCL Médecins Sans Frontiers student group	X	X	X

The focus group and interviews were recorded, and interview transcripts were sent to each interviewee for accuracy checks. All transcripts were then analysed thematically using atlas.ti [24]. In line with the exploratory and qualitative nature of the study, the data was interrogated using coding (open and analytical) and content analysis in order to identify themes for discussion [24, 25]. An initial set of themes were identified by the member of the research team who had conducted the focus group and interviews (NB). A second author (AI) also read the transcripts for key themes and any disagreements were discussed and resolved. The initial themes were then grouped into analytical themes for discussion amongst all of the authors. One of the authors (WB) conducted the initial literature review, which was used as the starting point of this paper, with the remaining authors subsequently identifying further relevant literature and developing the themes for discussion.

## Results

A number of interconnected themes emerged across the focus groups and interviews, and are discussed below, alongside illustrative extracts of the collected data. Although the second series of interviews was largely intended to gather student feedback on module (see Additional file 2), they also provided a space for further discussion of the ideas and themes initially discussed in the first series of interviews. The results below therefore treat the focus group and interview data as a single, amalgamated set.

The themes which emerged from this data centred both on issues that were specific to the field of global health and issues relating to the structure of the medical curriculum more generally. In particular, discussions focused on (1) conceptualisations of global health, (2) ideas about the relevance and legitimacy of global health issues to (i) students’ individual professional identities and careers, (ii) the medical curriculum and (3) perspectives on approaches to teaching and learning.

### Conceptualisations of global health

The data highlighted two key tensions related to students’ concepts of global health, which are explored below.

#### ‘Soft’ versus ‘hard’ knowledge

All students noted that global health includes broader topics than biological and clinical sciences and their associated competencies, but recognised that many of students would see these as ‘soft’ and therefore of less importance, as noted in the focus group discussion:

*S7: It’s seen as a soft subject that we don’t necessarily need to … whereas FGM [female genital mutilation] is like a medical thing that we might see in a clinic.*

*Interviewer: That’s very interesting, that somehow the soft [subjects] are lesser?*

*S7: Or you can get away with blagging them basically, I think that’s what it is. [general agreement from the group] If you were asked to write an essay on it we’d just sort of have a go, even if you didn’t really know much on it.*

*S5: It’s similar to what happened with Sociology in first year, a lot of people didn’t go to any of the lectures, thought they’d be able to blag it, didn’t get any marks, still passed the year… didn’t have to think about it ever again.*

For some respondents, wider social science knowledge and concepts such as public health, policy, and social determinants are fundamental to a good medical training and key to understanding health. S4 (uncertain) and S7 (advocate), for example, considered such topics to be essential for all medical professionals as healthcare is practised in a globalised world. For others, ‘soft’ social science subjects that explain the complex factors underlying health and illness are located lower down an imagined knowledge hierarchy, with a much stronger emphasis given to biomedical and clinical knowledge.

#### Local versus global

There were also evident tensions between concepts of global health as a ‘local’ versus a ‘global’ concern.

*“I think a problem maybe with the term itself is, when someone says ‘global health’ the first thing you think about is like Africa and like poorer countries, whereas that’s not necessarily true. Because you are considering the UK in the whole grand scheme of things, I think…” S6 [advocate; focus group discussion]*

Several participants noted that global health issues are part of contemporary medical practice in London, for instance due to the effects immigration on health and healthcare.

*“I think as much as people like to deny, we are … we’re very multicultural … I’m at a GP practice at the moment, obviously it’s a London practice, but more than 70% [of the patients] in this particular practice are from outside the UK. And so I think these things have a bearing on what you do even if you’re a [UK] GP” S4 [uncertain; interview 1]*

Others viewed global health as a specialist area of knowledge that can only be applied by professionals who intend to practise overseas.

*“It’s very difficult to relate to global burden of disease as cardiology reg in London. I mean it’s obviously something that anyone could see is important, but whether it’s you know relevant to you – I think it’s hard to like make people realise that it is, or to convince people that it is really.” S3 [sceptic; focus group discussion]*

#### Relevance of global health to the individual student

Students’ perceptions about the relevance of global health to their own professional development were linked to (i) their plans for the future and (ii) their previous experiences of global health teaching.

#### Future plans and identify formation

Opinion differed between those who saw global health as unrelated to their identities as doctors in the UK, and those who considered the lessons to have relevance regardless of where they practiced medicine.

*“I think for a lot of us, like the vast majority of us are going to work in London and you know if global health doesn’t apply in London where on earth does it apply?” S7 [advocate; interview 1]*

By contrast participant S3 considered it an unnecessary distraction, and felt that he represented a ‘silent majority’ of students who are uninterested but unlikely to speak up:

“*it’s very easy when someone sees an afternoon on Global Health just to not turn up”*.

As he explained later in the interview:

*“I mean most medical students in the UK will probably end up working most of their lives in the UK, and I’m not entirely sure it’s the greatest use of medical school time to be waxing lyrical about global health.” [sceptic; interview 1]*

(i) Previous experiences

Unsurprisingly, those who had already engaged with optional global health teaching or extracurricular activities in some form tended to be the strongest advocates for greater integration of these topics within the core medical curriculum.

The three students who self-identified as global health ‘advocates’ all had significant previous experience of the field, including through completion of the UCL Intercalated BSc in Global Health or through extracurricular work with non-governmental organisations such as Médecins Sans Frontières. The three students who self-identified as ‘uncertain’ either had no direct experience of global health (*n* = 2) or short-term experience through a student selected component (*n* = 1). The student self-identified as a ‘sceptic’ had no direct experience of global health beyond elements, such as the module which is the focus of this study, included in the core curriculum (see Table 2).

### Relevance of global health to the medical curriculum

The difference in perspectives in this area appeared to be linked to broader differences in understanding of the concept of ‘global health’ and what it covers. While some students viewed this as a specialist area only relevant to professionals who intend to practise overseas, others – such as S4 and S7 above – considered it to be an essential part of working in the globalised world and therefore relevant to all medical professionals.

Related to the issues above, the participants also discussed the importance of emphasising the clinical and scientific relevance of global health topics as a way of encouraging students to engage with it. This potentially also links to the discussion about the perceived importance of ‘soft’ vs. ‘hard’ subjects noted in the previous section.

*“Dry lecture series on social issues are definitely not going to engage medical students. If it’s possible to make it seem as clinically relevant as possible, that I think is the way to get the most engagement” S7 [advocate; interview 1]*

*“It’s hard to engage people who think it’s a waste of time before you start. Whereas, it’s much easier to see the value of stuff you find boring if it’s like, I don’t know, medically very relevant” S3 [sceptic; focus group discussion]*

*“… it’s a tainted term… like ‘Oh it’s global health – oh, don’t care’. Whereas if it’s like FGM, which is clearly a global health matter, then it’s… I think it’s just maybe about the way you advertise it.” S4 [uncertain; focus group discussion]*

### Perspectives on educational methods

Although interactive teaching styles were considered in part to contribute to the perception of global health as a ‘soft’ subject, it was also acknowledged that non-lecture based teaching methods were preferable for developing expertise in global health. To this end, group discussions, scenario-based work, and hands on experience were seen as most useful.

*“You only find it very interesting if you ... well in my opinion if you are getting hands-on experience, if you are actually seeing the impact of this work, rather than having a lecture about it” S2 [uncertain; interview 1]*

There were, however, significant doubts about the value of these approaches.

*“If I have a lecture from a specialist that’s an hour long and I look at the lecture notes, that is an hour of, you know, good information. Whereas if I have a discussion with eight of my peers facilitated by someone, then how do I know that what they’re saying is the correct thing?... it’s very hard as a medical student so worried about their exams; it’s hard to see the value with other people’s opinions.” S3 [sceptic; interview 1]*

## Discussion

The data from our small scale study highlights a range of diverse views among students regarding both concepts of global health and the intended outcomes of global health teaching. We suggest that these are underpinned by central issues related to the epistemology of contemporary medical knowledge and discourses. In particular, this includes understandings about which disciplines can or do legitimately inform medicine, and how the curriculum shapes or responds to emerging professional identities.

At one level, disagreement occurred in terms of the extent to which the students found global health reflected interesting and important concepts. Perhaps more significantly, however, were divergences of opinion between those who considered global health teaching relevant to medical practice in the UK and those who felt it to be a peripheral concern related to healthcare in ‘other’ places. While the intention of global health is to emphasise the interconnected nature of health issues around the world, (i.e. that it has global relevance, Rowson et al. [5]), the data suggests that there has not yet been a full shift away from the international health paradigm and that the message that global health is relevant to all doctors is often lost. It is noteworthy that those students who identified themselves as ‘advocates’ for global health had often not developed this commitment through academic study, but rather through involvement with organisations that were engaged in the practice of global health. This suggests the need to consider the extent to which the framing of global health teaching is successful in engaging those students who are not exposed to global health issues through other means.

Beyond interest (or lack of it) in global health learning, the structure of the medical curriculum and global health’s place within it may also present many barriers to effective student engagement. This was acknowledged by the advocate students as well as those who were uncertain or sceptical. At a fundamental level global health was not viewed as integral to the core demands of medical training. Rather, it was considered to be a ‘soft’ subject that struggles to compete with the demands of the ‘harder’ clinical and scientific elements of the medical degree. The position of global health within a pressured curriculum means that the efforts required to engage students are significant. We suggest that for students to be convinced of its importance to medical education, creative and innovative teaching methods need to be developed.

There is a risk, however, that certain teaching methods may have the unintended effect of further marginalising global health. While discursive teaching styles may be most appropriate in a normative sense, students in the study acknowledged that they can also serve to create a further separation between global health teaching and other parts of the medical curriculum. As a result of certain teaching methods global health may become more entrenched in the ‘soft’ side of teaching in contrast to the core curriculum where a more didactic approach is favoured.

From the perspective of the students in this study, the most effective methods for integrating global health training were to focus on topics where the clinical and scientific relevance was evident, in particular the relevance to medical practice in a multi-ethnic, socioeconomically diverse society. To be effective, they suggested that global health teaching needed to fit in with prevailing paradigms about what aspects of medical education and what forms of knowledge are most valuable.

### Limitations

There are a number of limitations to this study which should be acknowledged. Firstly, it is relevant to note that the study was carried out in the academic year 2014–2015 and is therefore reflective of the attitude of a group of medical students at that time. Given global events since then, it is possible that student levels of engagement in global health may have changed.

Secondly, as noted at the beginning of the paper, while the authors who designed the study (NB, AB and CW) had initially hoped for a larger group of study participants, this proved difficult to achieve given the demanding schedules of medical students. As a result, the study included a small and self-selected sample of volunteer respondents. It is likely that those students who already had strong feelings about global health were more willing to participate, which may have resulted in more diverse responses. The data and our conclusions are therefore difficult to make widely generalizable as the sample cannot be seen to reflect the views of the whole cohort of medical students. Nonetheless, by using qualitative approaches our work provides a useful exploration of student perspectives at the time of curriculum change.

## Conclusions

This study provides insight into student perspectives of global health teaching within the context of a global health teaching module that was integrated into a UK medical school core curriculum in the 2014–2015 academic session. We were in the process of launching a new core module – aimed at covering, with limited time and resources, key global topics – chosen because of their urgency and assumed relevance to all tomorrow’s doctors despite career intention or prior interest or experience.

The key finding from our data is that students are highly alert to the diverse epistemological issues that underpin global health. For some students, such interdisciplinarity is fundamental to understanding contemporary health and healthcare. For others, global health is merely a topic of geographic relevance. Furthermore, some students appeared to accept global health as a specialist area only relevant to professionals working overseas, while others considered it to be an essential part of working in the globalised world and therefore relevant to all medical professionals.

We argue that this second understanding is a more accurate depiction of the challenges that future medical professionals are likely to encounter in practice, particularly through work with diverse patient groups, due to the rapid global movement of both people and disease [26]. This suggests that more work needs to be done to explain the relevance of global health to medical students at the very beginning of their studies. There is a related literature on the positive impacts which global health teaching and learning can have on medical student’s perspectives which could be built upon. For example, Ibrahim et al. conducted a study of medical students’ perspectives following a teaching session on treatment of patients who were HIV-positive. Those students who had attended the session expressed a greater willingness to treat marginalized populations than those who had not [22]. Similarly, in an evaluation of a global health course at Harvard Medical School Nelson et al. found that 100% of students evaluated felt that they were better prepared to practice medicine in developing countries than previously [27].

Our data also supports previous quantitative studies which have found that experience of global health teaching is a significant factor in both increasing student support for global health within training overall, as well as in encouraging students to enter primary care medicine, obtain public health degrees, and practice medicine among marginalised populations [28]. A survey of graduates of the UCL BSc in International Health in 2011 (the title of which was changed to Global Health in 2012) found that significantly higher numbers of students who had done the BSc were choosing careers in public health (12%, compared to the UK average of 0.8%) and paediatrics (10%, versus the UK average of 3.2%) [29].

However, challenges to integrating global health into the core medical curriculum exist at two key levels. In addition to some students’ perceptions about the lack of relevance of global health to their training, as discussed above, the structure of the medical curriculum does not lend itself to the incorporation of global health teaching, where it comes into conflict with those subjects that are central to clinical practice, taught in a didactic manner, and subject to examination. In order to most effectively engage students in global health, teaching must fit in with prevailing norms about the sort of knowledge that is valued in medical education, and clinical and scientific relevance of any global health topic must be emphasised.

Finally, we recognise that the training of doctors does not remain static – what is core, what is optional, what is taught and assessed, and how, needs to adapt to constantly emerging healthcare challenges. Making changes such as these raises significant questions for educators, including how to engage students in new topics. As educators and advocates for a greater understanding of global health, we could be deterred. However, we suggest an urgent need to attend to this diversity in our pedagogic practice.

## Additional files

Additional file 1: Focus Group Session Plan – Provides an outline of the information provided at the start of the session as well as guiding questions for the discussion. (DOCX 17 kb)

Additional file 2: Interview Guides – guiding questions for individual interviews with students both at the beginning of the project and again three months later after the teaching intervention was finished. (DOCX 17 kb)

## Data Availability

In order to protect the confidentiality of research participants, the data generated and analysed during the current study are not publicly available.
