# Supplementary material for: Medical students as global citizens: a qualitative study of medical students’ views on global health teaching within the undergraduate medical curriculum
Source: BMC Med Educ. 2019 May 30;19:175. doi: 10.1186/s12909-019-1631-x (PMC6543666; doi:10.1186/s12909-019-1631-x)
Supplement: Supplementary file 1 — Focus Group Session Plan – Provides an outline of the information provided at the start of the session as well as guiding questions for the discussion. (DOCX 17 kb) [file 12909_2019_1631_MOESM1_ESM.docx]

**Appendix 1: Focus Group Session Plan**

**Introduction**

- Overview of the project
- Aims of this session
- Confidentiality
- Recording
- Right to withdraw at any time (and how to contact me)
- Questions?

**Guiding questions for discussion:**

- What words do you associate with the term ‘global health’? Does it convey positive or negative images to you? (Brainstorm with flipchart)
- Do you think that global health should be taught as part of the core MBBS curriculum at UCL? Why or why not?
- If global health was part of the MBBS curriculum, what should it contain? What global issues do you think all medical students need to know about, if any?
- How do you think that global health should be taught? (e.g. lectures, group work, overseas electives, placements in London NGOs?)
- Have a look at these proposed learning outcomes (PPT slide of Johnson et al (2012)). Do you think that all medical students need to know these things? Why? Why not?
- Are you aware of UCL’s Global Citizenship Strategy? If so, do you think the Medical School global health teaching relates to it in any way?

**Conclusions**

- Is there anything else you want to say at this point re: global health teaching at UCL?
- I will be in touch about follow-up discussion/ interviews if anyone is happy to take part.
- Thanks for your participation!
