# Supplementary material for: Medical students as global citizens: a qualitative study of medical students’ views on global health teaching within the undergraduate medical curriculum
Source: BMC Med Educ. 2019 May 30;19:175. doi: 10.1186/s12909-019-1631-x (PMC6543666; doi:10.1186/s12909-019-1631-x)
Supplement: Supplementary file 2 — Interview Guides – guiding questions for individual interviews with students both at the beginning of the project and again three months later after the teaching intervention was finished. (DOCX 17 kb) [file 12909_2019_1631_MOESM2_ESM.docx]

**Appendix 2: Interview Guides**

**Guiding questions: semi-structured interviews (phase 1)**

- Can you say a bit more about yourself and how you came to choose medicine as a career? What do you think you will specialise in?
- Do you have any previous study/ work/ personal experience related to global health?
- Where would you place yourself on a continuum that ranges from ‘enthusiastic’ to ‘unconvinced’ when it comes to global health?
- Have you taken part in any global health teaching as part of the MBBS curriculum in the past? What did you think of it?
- Do you think that global health should be taught as part of the core curriculum? Why or why not?
- Are there any specific global health topics that we talked about in the focus group (e.g. vaccine programmes, water, gender inequality, etc.) that you think should be covered within global health teaching or in any other part of the curriculum?
- What types of teaching and learning styles do you think would work well with these kinds of global health issues/ topics? (e.g. lectures, overseas electives, group work, NGO placements) Why?
- How do you think global health teaching at UCL could be improved overall?

**Guiding questions: semi-structured interviews (phase 2)**

- What did you think of the global health module as a whole?
- What did you think of overall organisation/ structure of the module?
- Which teaching and learning approaches did you like or not like? Which would you be happy to use again?
- What did you think of the tutors? Did they seem enthusiastic? Knowledgeable?
- Did you feel the global health topics were covered well?
- How would you like this to be followed up in later years?
